# Gamma Radiation Processed Polymeric Materials for High Performance Applications: A Review

**DOI:** 10.3389/fchem.2022.837111

**Published:** 2022-03-14

**Authors:** Amol Tarachand Naikwadi, Bhuwanesh Kumar Sharma, Keyur D. Bhatt, Prakash A. Mahanwar

**Affiliations:** ^1^ Department of Polymer and Surface Engineering, Institute of Chemical Technology, Mumbai, India; ^2^ Department of Chemistry, Faculty of Science, MUIS, Ganpat University, Mehsana, India

**Keywords:** gamma radiation induced grafting, gamma induced crosslinking, polymer modification, high performance applications, electron beam radiation

## Abstract

The polymeric properties are tailored and enhanced by high energy radiation processing, which is an effective technique to tune the physical, chemical, thermal, surface, and structural properties of the various thermoplastic and elastomeric polymeric components. The gamma and electron beam radiation are the most frequent radiation techniques used for crosslinking, compatibilizing, and grafting of various polymer blends and composites systems. The gamma radiation-induced grafting and crosslinking are the effective, rapid, clean, user-friendly, and well-controlled techniques for the polymeric materials for their properties improvement for high performance applications such as nuclear, automobile, electrical insulation, ink curing, surface modification, food packaging, medical, sterilization, and health-care in a different environment. Similarly, electron beam radiations crosslinking has been a well-known technique for properties development and has economic benefits over chemical crosslinking techniques. This review focuses on the development of polymeric multi component systems (functionalized polymer, blends, and nanohybrids), where partially nanoscale clay incorporation can achieve the desired properties, and partially by controlled high energy radiations crosslinking of blends and nanocomposites. In this review, various investigations have been studied on the development and modifications of polymeric systems, and controlled dose gamma radiation processed the polymer blends and clay-induced composites. Radiation induced grafting of the various monomers on the polymer backbone has been focused. Similarly, comparative studies of gamma and electron beam radiation and their effect on property devlopment have been focused. The high energy radiation modified polymers have been used in several high performance sectors, including automotive, wire and cable insulation, heat shrinkable tube, sterilization, biomedical, nuclear and space applications.

## 1 Introduction

It has been more than 30 years since researchers began potentially exposing polymers to high energy radiation such as gamma and electron beam radiation for crosslinking, grafting, and compatibilization (Clough, 2001; Senna et al., 2010; Ashfaq et al., 2020). Radiation-treated polymeric molecules have established their importance in the global market for various applications (Tamada and Maekawa, 2010). Since the last 3 decades, radiation processed polymeric multi-components such as functionalized polymer, polymer blends, and composites have been widely used in automobiles, constructions, aerospace, nuclear, defense, electrical, electronic, high temperature applications, etc. (Clough, 2001; Cleland et al., 2003; Bhattacharya and Misra, 2004). The ionizing radiations such as gamma and electron beam play an essential role in the modification of various industrial polymers (thermoplastics and elastomers) such as low-density polyethylene (LDPE), high density polyethylene (HDPE), Nylon-6, Nylon-6 6, ethylene-propylene-diene monomer (EPDM), polyolefin elastomer (POE), silicone elastomer, ethylene-vinyl acetate (EVA) copolymer, etc. (Chowdhury and Sabharwal, 2011; Ray Chowdhury et al., 2016; Sharma et al., 2018). The gamma radiation ionizes the polymeric chain, leading to chain crosslinking and scission via a free radical mechanism. The degree of crosslinking depends upon polymer structure, phase morphology, irradiation of gamma radiation with controlled dose and duration, and nature of gamma radiation source (CHARLESBY, 1967; Radiation Technology in Emerging Industrial Applications, 2000). In general, the radiation-induced ionization process occurs in two stages. The first stage involves the breakage of covalent bonds, which decompose into free radicals. The generated ions then cause chemical interactions between the molecules at varying concentrations in the second phase (Kudoh et al., 1996; Sonnier et al., 2016; Ashfaq et al., 2020).

The gamma radiation exposure of polymers has become a common technique for structural modification, polymerization, grafting, sterilization, and crosslinking of various thermoplastic and elastomers (Erizal et al., 2015; Ashfaq et al., 2020). The new products made by gamma radiation processing are indeed valuable and competent materials for high performance applications (Sirkar and Ho, 1992). The research and investigation in polymer radiation technology have opened the door for high performance applications with high commercial and economic importance in the packaging, automotive and electronic sectors (Rouif, 2005).

The gamma radiation crosslinking and surface modification of polymers enhance their mechanical, thermal, chemical, electrical insulation, and environmental properties, which finds them suitable for high performance applications, e.g., space, automobile, constructions, nuclear, and defense applications (Bhattacharya, 2000; Clough, 2001; Cota et al., 2007; Tamada and Maekawa, 2010). Radiation dose is nothing but the amount of energy absorbed per kilogram of polymeric material. This radiation dose is measured in kiloGray (kGy) (Sonnier et al., 2016). Commonly, polymers possessing rigid and aromatic structures show high energy radiation and temperature resistance properties. However, some elastomers don’t have aromatic structures but can withstand ionizing radiation due to their amorphous and coiled structure (Sharma et al., 2018). Some of the polymers and polymeric blends may degrade on this dose range. Most polymers required radiation dose frequency between 15 and 200 kGy. These polymers include polypropylene (PP), polyvinyl chloride (PVC), butyl rubber and cellulose, etc. (Smirnov et al., 2009; Al Naim et al., 2017).

Both polymer blends and composites are playing their role in attracting the attention of industrialists, academicians, researchers for a wide range of applications (Muhammad et al., 2021) (White et al., 2013) (Keizo Makuuchi, 2012) (Singh et al., 2010; Settings et al., 2016).

The high energy processed polymers are gaining popularity for wide areas of high performance application due to several advantages after irradiation of polymers. Radiation crosslinked polymer at optimised dose demonstrate better property development compared to chemical crosslinking. As radiation processing is more convenient, clean, toxic free and environment friendly compared to peroxide crosslinking and ethylene oxide sterilization. Different polymeric systems can be irradiated by electron beam with controlled and different doses in the large capacity while chemical crosslinking of polymer takes long time for processing of polymer in large capacity which might cause also health hazardous issues to human. Radiation crosslinked polymer at controlled doses show numerous advantages of property development such as excellent mechanical strength, high thermal insulation, electrical insulation, chemical resistance, radiation resistance, anti-microbial property, and environmental stress cracking resistance for long term durability[*Singh and J. Silverman (Eds.), 1992, Reichmanis et al.;1993*]. Due to excellent improvement in required properties, polymeric material such as thermoplastic, elastomers, thermoset resin, liquid resin, emulsion, paint and ink materials are considered for high performance applications (Shalaby and Clough, 1996; Clough and Roger, 2022). The crosslinked polymeric products are considered on priority basis for sector wise different end use applications e.g. automobile sector, wire and cable insulation, heat shrinkable tube, surface anti-microbial coating, cured ink, emulsion for adhesion, food packaging, hydrophobic cotton fabric, defence and nuclear sector, gasket, seals and O-ring, health care, biomedical (tissue engineering, drug delivery and hydrogel) and sterilization for their excellent performance in desired properties [Clough, R., 2001, *W. Knowlle, C. Trautman (Eds), 1999*].

The high temperature polymers are expected to have a continuous service temperature of more than 100°C. Reinforcements and fillers (metal oxides) add strength and incorporate high temperature resistance properties to the polymer matrix. This review majorly focuses on gamma radiation modified polymers for high performance applications. This review is divided into parts related to the gamma radiation modified polymers, the effect of radiation on polymer properties, the discussion of different polymer blends and composites, and the high performance applications of the radiation modified polymers.

## 2 Different Radiation Processes for Polymer Modifications

Polymer grafting, crosslinking, and sterilization can be achieved using chemical processes using different additives. These grafting, crosslinking, and sterilization reactions required additives to initiate the reactions. These types of additives are toxic in nature, environmentally harmful, costly, etc. Radiation processing polymers have benefits over chemical processes as no additives are necessary for the reaction, can be used at any temperature range applications, well controlled grafting and crosslinking, etc.

Irradiations of polymers are mainly divided into alpha radiation, X-ray photons, electron beam radiation (E-Beam), and gamma radiation (**γ-**R) as high energy solid state processes. Many researchers have studied the irradiation process and mechanical milling to mix and compatibilized the immiscible blends (Smith et al., 2001). E-Beam and **γ-**R are compared based on the different dose rates and penetration depth. Many researchers have reported that **γ-**R has a greater penetration depth. The primary modification and product development using alpha radiation, electron, and gamma induced polymer modifications are identical. However, alpha radiation differs from E-Beam and **γ**-R due to the difference in dose ranges, the formation of radicals, and the efficiency of additives in alpha radiation (CHARLESBY, 1967; Ashfaq et al., 2020). The deposition of energy can be done through columbic interactions and high energy photons, which take place in the range of 10^–10^–10^–12^ s. This is the approximate range of ionized and excited compounds generated alongside the tracks (Ashfaq et al., 2020). In the last 5 decades, researchers have developed and studied potential properties and practical high performance applications on irradiated polymers as well as the effect of radiation on different polymeric reactions (Reichmanis et al., 1993; Dahlan, 1994; Gueven, 2004; Shinyama, 2018).

In comparison with E-Beam radiation, **γ**-R has higher penetration power. The negative charge particles of E-Beam radiation limit its penetration power. The comparative studies of E-Beam radiation γ-R have been shown in Table 1.

**TABLE 1:** Differentiation of gamma radiations and electron beam radiations (Dahlan, 1994).

Sr. No	Characteristics	Electron beam	Gamma radiation
1	Energy	1.17 + 1.33 MeV	Variable 0.2–10 MeV
2	Power	1.48 kW/100 kCi	Variable 4–400 kW/unit
3	Dose rate	Low (Mrad/hr)	High (Mrad/sec)
4	Penetration	High (43 cm in water)	Low (−0.35 cm/MeV)
5	Energy utilization efficiency	Low (−40%)	High (−90%)
6	Production rate	Low	High
7	Maintenance	Replenishment of Co-60 source	Replacement of electronic parts
8	Others	Source decay 1%/month	Shut-off power source

**TABLE 2:** Gamma irradiated polymer blends and composites for high end applications.

Sr. No	Polymeric system	Property investigated	Application	References
1	PP + Sisal fibre and wood flour composite	Gamma irradiation effect (10–70 kGy) on mechanical. thermal and morphological properties of composites	Construction application	Albanoa et al. (2002)
2	Gamma grafted LDPE-clay nano composite	Mechanical, Thermal, Crystallography and Morphological properties	High performance application, automobile and construction	Chem et al. (2011)
3	TPE + CNT and MMT nanocomposite, Blend of TPE composite with PLA and NR	Gamma irradiation effect (0–250 kGy) on mechanical, thermal, and conductivity of the nanocomposite	Gamma modified nanocomposite for electronic packaging application	Tarawneh et al. (2021)
4	Sepiolite filled EPDM composite	Effect of Gamma irradiation by 50 kGy on mechanical, crosslink density, thermal stability, and morphological behavior of EPDM composites	High temperature rubber composite application	Mohd Zaini et al. (2022)
5	Gamma radiation resistant EVA/EPDM blends with variation in VA content in EVA.	Effect of Gamma ageing Mechanical, compression set, thermal and morphological properties of EVA/EPDM blends by 500, 1,000, and 1,500 kGy	High temperature and Gamma radiation resistant materials (Gasket, Seal and O-ring)	Sharma et al. (2018)
6	Gamma-ray modified UHMWPE-Clay (Magnesium silicate hydrous) Composites	Effect of Gamma crosslinking by 25 and 50 kGy on crosslink density, Oxygen index and Crystallinity	Medical implants, defence armor, and bulletproof jackets	Bakhsh, (2021a)
7	Glass fibre and Carbon cloth reinforced PE, PP and polyamide (PA) composite	Study of gamma radiation resistance of different thermoplastic matrix composite (PE, PP and PA) mechanical and thermal properties. (50–250 kGy)	High temperature and thermal resistant composite materials	Smirnov et al. (2009)
8	LDPE + Carbon black loaded in different concentrations	Effect of gamma crosslinking on mechanical and physio-chemical properties of LDPE-carbon black composite	Film packaging for food preservation application	Salem et al. (2003)
9	Thermoplastics such PE and PP composites sisal fibre and wood flour. Blend of Styrene-butadiene rubber with PE, PP and PA-6	Effect of gamma irradiation on the functionalization of PE and PP for blend compatibility with SBR and composite with fibre	Biomedical and packaging application	Albano et al. (2002)
10	Blending of oxidized ground tyre rubber powder (GTR) + LDPE and HDPE	Gamma and KMNO_4_ oxidation treatment of GTR powder and blend compatibility with MA-g-LDPE. Studies of mechanical, chemical and morphological properties	Automobile and commodity applications	Sonnier et al. (2007)
11	Radiation grafting of monomer 2-hydroxyethyl methacrylate (HEMA) on the LDPE chain in the absence and presence of air	The gamma radiation grafted samples were characterized by thermal analysis techniques (DSC and TGA) and by Fourier transform infrared spectroscopy (FTIR)	To increase the hydrophilicity of grafted LDPE film for bio applications	Ferreira and Gil, (2005)

**TABLE 3:** Difference between gamma and electron beam radiation processing of polymeric Materials.

S.No	Criteria	Gamma ray processing	Electron beam (EB) processing
1	Source	Cs-137 or Co-60	Electron accelerator
2	Energy (MeV)	1.17–1.33	0.5–12 (keeps variation)
3	Polymer degradation	Chain degradation is higher compared to chain crosslinking in most thermoplastics matrix	Chain crosslinking is higher in most thermoplastics matrix
4	Penetration depth	Gamma irradiation can be processed upto higher (14 mm) depth of thickness	Electron beam irradiation cann’t be processed for material of higher thickness
5	Irradiation time	Low speed and dose rate	High speed and exposure at different dose rate
6	Capacity	Gamma processing possesses low volume due to small chamber capacity	EB has larger volume and capacity for irradiation
7	Hazardness	Gamma processing is more hazardness due to radiactive decay and higher energy	EB processing has been observed clean, more durable, and environmental friendly in comparison to gamma processing
8	Applications	Monomer grafting, hydrogel synthesis, medical device strerlization and polymeric nuclear devices	Wire and cable insulation, heat shrinkable tube, sterilization, compatible thermoplastic-elastomer blends, automobile and cured ink and antimicrobial coating
9	Material degradation	High probability of product damage	Reduced product damage
10	Processed materials	LDPE/EPDM, PP/HDPE, PP-clay composite, PP-medical syringe, EVA/EPDM, PP-sisal and glass fibre composite and HDPE-GTR composite. Monomer grafting on polyolefin based elastomer	LDPE/EPDM blend EVA/EPDM blend, LDPE-nano clay composite, PP/EPDM blend, LDPE/EVA, EPDM-nano clay composite

## 3 Property Development by Gamma Radiation (γ-R) Modification

**FIGURE 1:**
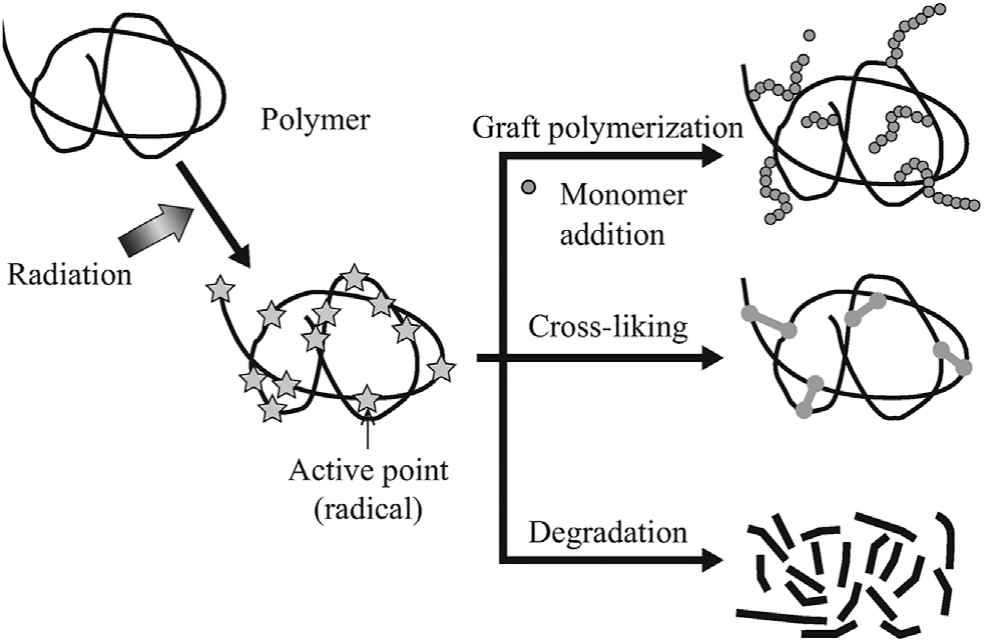
The effect of high energy (gamma) irradiation on polymers in different manner (Tamada and Maekawa, 2010).

Gamma radiation (γ-R) is basically electromagnetic radiation occurred when an unstable atomic nucleus loses energy. This type of radiation consists of the highest amount of proton energy and delivers numerous practical high performance applications. Both chain crosslinking and chain scission occur simultaneously in the polymer matrix in gamma irradiation. The gamma radiation crosslinked polymers in a controlled dose manner (1–10 kGy) are capable of withstanding high energy gamma radiation and high temperature environment with retention of their useful properties (Kudoh et al., 1996; Rouif, 2005; Fel et al., 2016). By subjecting the polymer product to high energy ionizing radiation as well as temperature for a set amount of time, it should be able to maintain its product and reliability properties at specified operational conditions. The gamma radiation processed polymer blends and composites can withstand high energy radiation and temperature while retaining relevant mechanical, thermal, thermo-mechanical, physio-chemical, and environmental properties (More et al., 2021). Figure 1 presents the effect of gamma radiation on different types of polymer reactions.

The gamma radiation modification imparts the following developments in the polymeric system, e.g., thermoplastic, elastomer, thermoplastic elastomer blends, and nano-composites:

➢ The degree of compatibility in thermoplastic-elastomer, elastomer-elastomer blends to improve mechanical, thermal, morphology, and environmental properties
➢ The crosslink ability to improve mechanical, thermal, chemical resistance properties.
➢ The high energy radiation resistance property without deterioration of physical shape, mechanical, thermal, and environmental properties
➢ The high temperature resistance property without any alternation in color, physical shape, and dimensional stability
➢ The preparation of hydrogels for medical and health care applications.

Albano et al. investigated the thermal and mechanical properties of PP composites made with sisal and wood fibers using gamma radiation at a dosage rate of 4.8 kGy/h at room temperature. The gamma radiation also results in irreversible changes in the thermal and mechanical properties of the polymers. This can be due to the result of chain scission, crosslinking, high energy irradiation, shattering of certain covalent bonds, and release of active free radicals (Clough, 2001; Hazarika et al., 2012; Tayel et al., 2015). Ashhab et al. have discovered the effect of gamma radiation on the cellulose acetate polymer. They have claimed that radiation exposure time influences cellulose acetate’s chain scission and intrinsic parameters with varying radiation doses (El-ashhab et al., 2013).

### 3.1 Gamma Induced Grafting of Monomer on the Polymer Backbone

**FIGURE 2:**
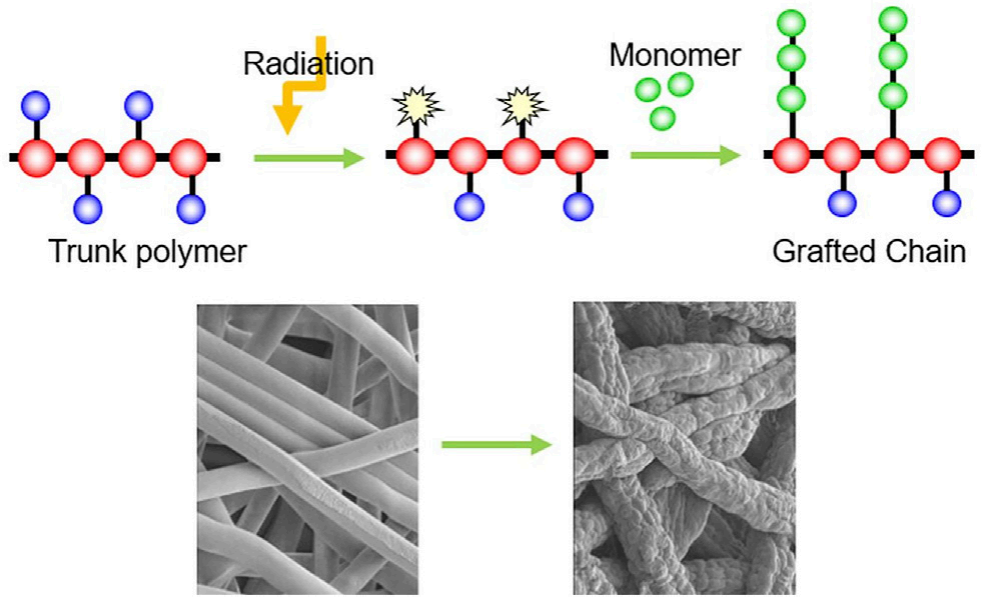
Simplified structure of radiation grafted polymer.

Gamma radiation-induced grafting and crosslinking are efficient, fast, clean, user-friendly, and well-controlled techniques for improving the properties of polymeric materials for nuclear, automobile, electrical insulation, construction, medical, and health-care in a variety of environments (Rouif, 2005). Radiation-induced grafting is a valuable and common approach for grafting of polymers. Gamma radiation doses in the range of 1–30 kGy are the most commonly employed for the grafting technique, with an efficient dose rate of 1–3 kGy recorded. The gamma radiation grafting approach is preferable to the physical or thermal induced processes since it does not require any additional additives for grafting (Ramos-Ballesteros and Lo, 2016; Walo, 2022). Figure 2 shows the simplified structure of radiation grafted polymer.

**FIGURE 3:**
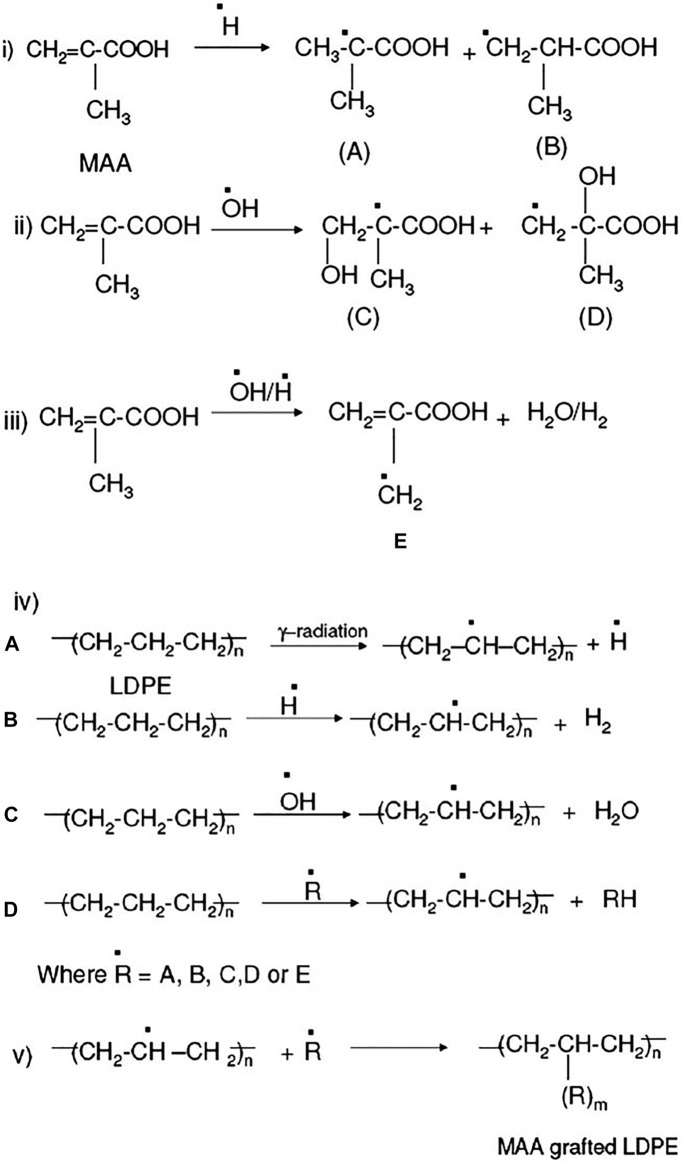
Gamma radiation induced grafting on low density polyethylene (LDPE) (Chowdhury and Sabharwal, 2011).

In recent years, researchers have been focusing on the irradiation of polymers. Since this gamma radiation induced grafting approach is less damaging, less poisonous, more environmentally friendly, and takes less time than traditional grafting techniques. The functionalization approach has primarily been utilized to establish compatibility in polymers. This improves conventional chemical grafting on polymer backbones, such as maleic acid, maleic anhydride, glycidyl methacrylate (GMA), and acrylamide, which acts as compatibilizers in the blending process (Carianni, 1998; Kim et al., 2002; Covas et al., 2011; Fu et al., 2020). Chowdhury et al. have grafted the methacrylic acid (MAA) on the LDPE polymer chain using gamma radiation to increase the compatibility with organic clay by lowering the hydrophobicity of LDPE (Chowdhury and Sabharwal, 2011). The functionalization and grafting of MAA on LDPE have shown in Figure 3. There is much literature reported on chemically grafted polymer compatibilizers. Most investigators have used this conventional technique to establish the compatibility between two polymer phases, as shown in Figure 3.

Gargan et al. used this gamma irradiation technique to graft hydrophilic monomer onto polyolefin polymer. In this work, the authors have grafted the acrylic acid and methacrylic acid onto pre-irradiated LDPE (GARGAN et al., 1990). Kornacka et al. proposed the effect of gamma irradiation on polymer surface functionalization for the separation of heavy metal ions. A 10 kGy dose was used to graft acrylic acid and acrylamide on a polypropylene filter. The gamma irradiation was used to functionalize the PP filter surface, followed by acrylic acid and acrylamide grafting onto the PP backbone chain. The prepared copolymer was then used to preconcentrate and eliminate the metal ions, including lanthanides from waster water (Kornacka et al., 2014).

### 3.2 Development of High Performance Nano-Clay Hybrid Using Gamma Radiation Grafted Compatibilizer

Gamma radiation grafting of a polar group on a polymer chain is a fast and environmentally friendly physical procedure that produces no contamination, unlike chemical grafting. Radiation grafting does not necessitate the removal of any chemical or by-product. In this technique, functional groups are grafted on the surface only, without affecting the main polymeric matrix. An idea has been employed to change the hydrophilic–hydrophobic property of LDPE by grafting the polar group of methacrylic acid (MAA) on the surface of LDPE by gamma radiation. Only 10 wt.% of MAA-g-LDPE with 90 wt.% un-grafted LDPE has been used to synthesize the LDPE-based hybrids (Chowdhury and Sabharwal, 2011). Tarawneh et al. have developed the nano composite of thermoplastic elastomer with carbon nanotubes and montmorillonite. They have studied the effect the gamma radiation on different mechanical, conductivity, and thermal properties of the nano composites. The thermoplastic elastomer was prepared to form polylactic acid and natural rubber based polymeric blend. They had varied and studied the gamma radiation dose range in the range of 1–200 kGy and reported that the composites’ significant properties were achieved when the dose range increased to 150 kGy (Tarawneh et al., 2021). Samaa et al. have prepared nano composites of the Ethylene propylene diene monomer rubber (EPDM) with nano clay by gamma radiation process. The nano clay has been used to increase EPDM’s resistance to gamma radiation.

#### 3.2.1 Gamma Radiation Grafting of Metha-Acrylic Acid on Low Density Polyethylene Chain

A polar group, methacrylic acid (MAA), was grafted onto LDPE by gamma radiation to increase the compatibility of LDPE with organically modified clay by reducing the hydrophobicity of LDPE. Methacrylic acid (MAA) is grafted on the surface of the LDPE by gamma radiation in the presence of aqueous ammonium ferrous sulfate [(NH_4_)_2_Fe(SO_4_)26H_2_O)] by the direct method. Ammonium ferrous sulfate was used to inhibit homo-polymerization. The sample was then irradiated with a total dose of 1.67 kGy at a dose rate of 2.5 kGy/h using a Co-60 radiation source in the gamma chamber. It was seen that the degree of grafting of MAA on LDPE is around 5 to 7 wt% at 1.67 kGy doses.

#### 3.2.2 Preparation of High Performance MAA-G-LDPE-Clay Nano-Hybrid

**FIGURE 4:**
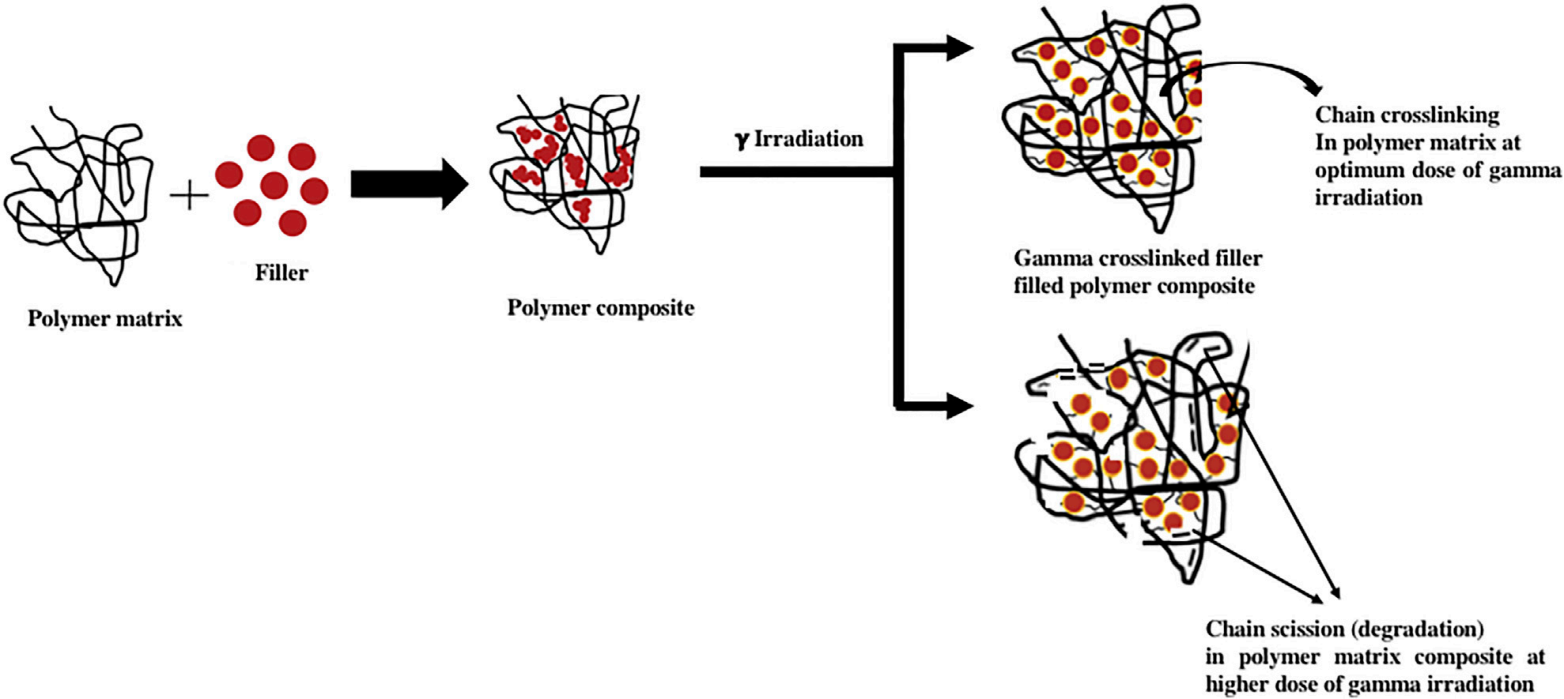
Schematic diagram of gamma irradiation chain crosslinking and scission phenomenon in filled reinforced polymer composite.

Synthesis of nanohybrids 10 wt.% g-LDPE, 90 wt.% LDPE and various amounts of clays (20A: 2, 5, and 8 wt.%) were mixed for 4 min in a Brabender platicorder at 140°C at a rotor speed 50 rpm. Specimens were characterized to justify the compatibility between the MAA-grafted LDPE matrix and nano-clay. Figure 4 shows Schematic diagram of gamma irradiation chain crosslinking and scission phenomenon in filled reinforced polymer composite.

### 3.3 Gamma Radiation Modified Polymer Composites

**FIGURE 5:**
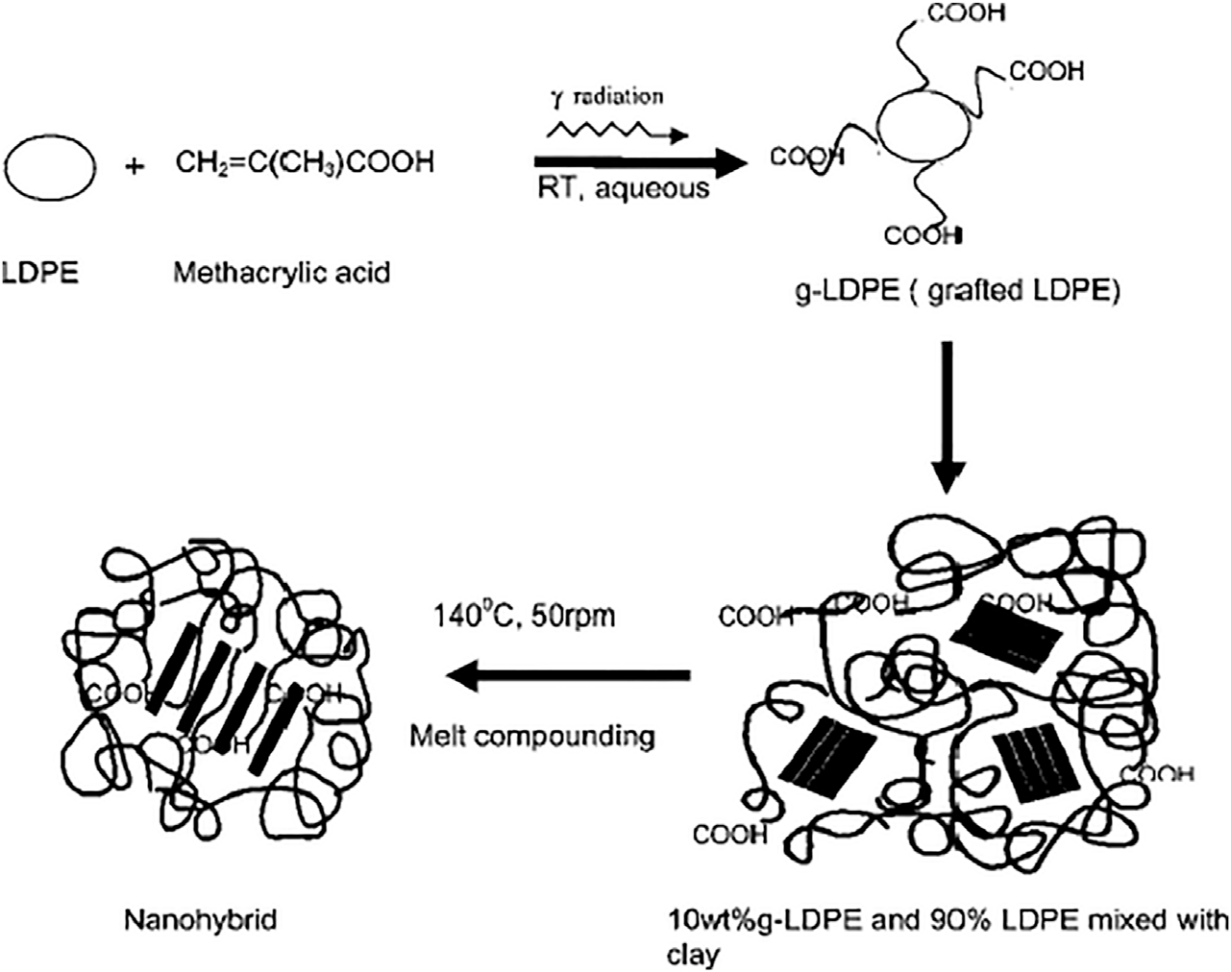
Schematic diagram of gamma grafted MAA-g-LDPE and nano clay composite.

Several researchers have been studied the effect of gamma irradiation on different organic and inorganic loaded polymeric materials. They have also explained the effect of irradiation on the mechanical, chemical, and thermal properties of polymeric composites. Commodity polymers such as low density polyethylene (LDPE), high density polyethylene (HDPE) and linear low density polyethylene (LLDPE), and polypropylene (PP) are being used in several household applications. The engineering applications of such polymers are limited due to their lower mechanical properties. Preparation of blends and composites of polyolefin has resulted in higher thermo-mechanical properties. Salem et al. have revealed the effect of gamma irradiation on carbon black loaded LDPE polymeric films. They have investigated the different gamma irradiation doses and different carbon black concentrations on thermo-mechanical properties of LDPE films. The authors have presented the changes in properties of LDPE films due to high irradiation and showed the correlation between oxidative chain scission and crosslinking. The gamma irradiation in the range of 5–30 kGy was utilized to crosslink the LDPE films. They have observed that LDPE film composite with 7 wt.% carbon black loading showed stabilized tensile properties and can be used for packaging applications (Salem et al., 2003). Figure 5 shows Schematic diagram of gamma grafted MAA-g-LDPE and nano clay composite.

Smirnov et al. have investigated the effect of gamma irradiation on polymer composites based on glass and carbon cloth fibers. They have studied several polymer matrices like polyethylene (PE), polyamide (PA), and polypropylene (PP) and prepared the polymer composite materials reinforced with glass and carbon cloth fibers. They have observed that the thermoplastic matrix composite of PP and PA shows lower radiation resistance than the PE polymer matrix composite. The thermoplastic matrix based composites were prepared using gamma radiation doses in between 50 and 250 kGy. It was observed that carbon cloth reinforced PE composites show lower radiation resistance than that glass reinforced PE composites. They have also studied the effect of gamma irradiation doses onto polymer composite properties for the application in engineering sectors (Smirnov et al., 2009).

Several researchers have studied the effect of gamma radiation on polymeric blends and their nano-micro composites. Albano et al. have studied the blends of different polyolefins, polyamides, and styrene polymers. They have used gamma radiation to functionalize the polyolefin to generate active sites onto the polymer backbone. Further, the prepared polymer blends are mixed with different fillers, and gamma radiation was used to promote the interaction between polymer-filler segments. The activation energy was observed to be changed with varying gamma radiation doses (Albano et al., 2010).

Recently Bakhsh et al. have analyzed the effect the gamma radiation on polymeric composite properties. They have fabricated the composites of ultrahigh molecular weight polyethylene (UHMWPE) reinforced with magnesium silicate hydrous (sepiolite) filler. The sepiolite filler was used in different concentrations along with varying doses of gamma radiation. The composites have been fabricated and irradiated with 25 kGy, and 50 kGy gamma radiation doses and the maximum concentration of sepiolite filler used is 3 wt.%. UHMWPE irradiated composite can be studied industrial scale for medical implants, defense armor, and bulletproof jackets (Bakhsh, 2021b).

### 3.4 Gamma Radiation Crosslinked Polymeric Blends

**FIGURE 6:**
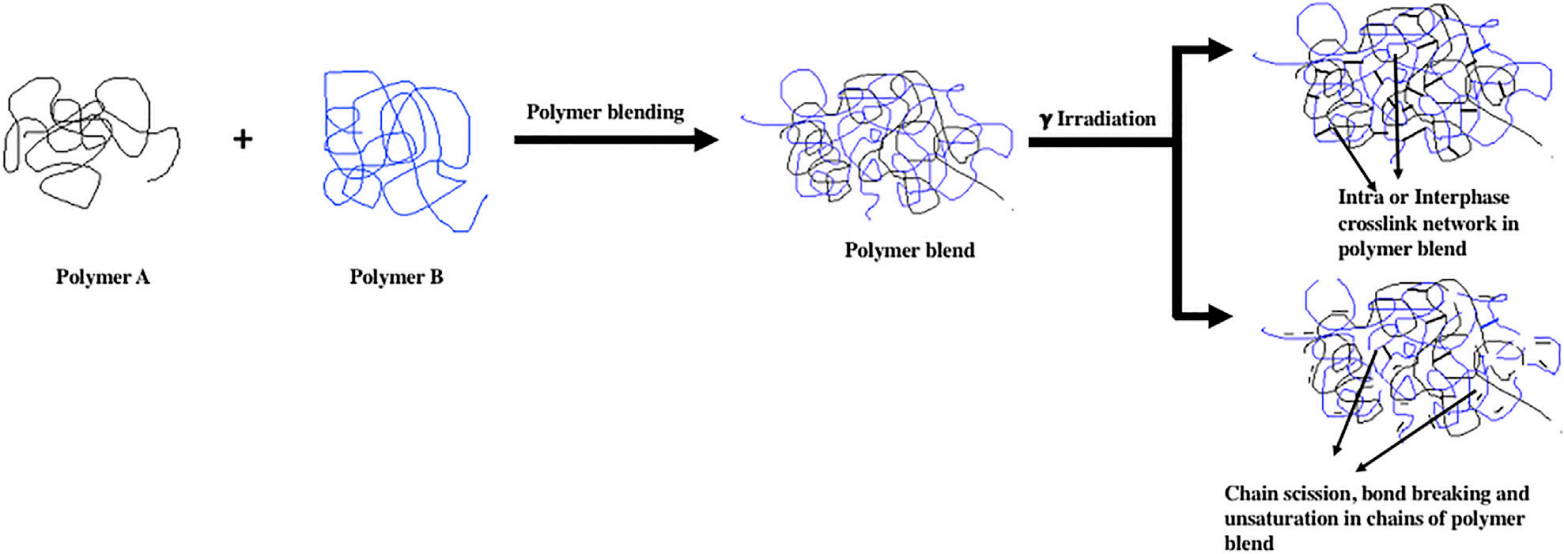
Schematic diagram of the effect of gamma irradiation on chain scission and chain crosslinking in intra and intraphase in polymer blends.

Figure 6 shows Schematic diagram of the effect of gamma irradiation on chain scission and chain crosslinking in intra and intraphase in polymer blends. Polymer blends are prepared to enhance polymer properties for high end applications. There are different techniques employed to prepare polymer blends, such as extrusion melt blending and solution blending. Efficient enhancement in the polymer properties can be achieved after crosslinking or polymer networking. Gamma radiation in controlled dose also establishes the interface compatibility in incompatible polymer blends. Gamma crosslinking depends on polymer morphology, where the amorphous phase shows a high formation of the crosslinked network compared to the crystalline phase. Since the amorphous phase has a random, coiled structure and free mobile segment, which enables the matrix for ionization and free radicals among chains that recombine to generate a 3D crosslink network. Researchers have found that gamma radiation induced polymer and polymer blends crosslinking can improve the properties and be well-suited for high end applications. Several researchers have investigated the elastomer and thermoplastic polymer blends and curing using gamma radiation. Hassan et al. have studied the effect of gamma radiation and dicumyl peroxide on the curing reaction of devulcinised rubber and polypropylene blends. They have prepared the thermoplastic vulcanizates of devulcinised rubber and polypropylene with a weight ratio of 75:25. The irradiation observed doses were used as 25, 50, 75, and 100 kGy with a radiation dose rate of 4 kGy/h. They have found that mechanical and thermal properties of the irradiated polymer blend networks possess higher properties than unirradiated polymer blends (Hassan et al., 2014). Ashok et al. have developed the EPDM and chlorobutyl rubber (CIIR) blends and crosslinked with gamma radiation for gaskets and o-rings in nuclear applications. The main objective of this research was to overcome the demerits of EPDM, which was poor hydrocarbon resistance, and to improve the application life of EPDM in the nuclear environment. The EPDM/CIIR blends were irradiated at cumulative doses of 0.5, 1, and 2 MGy with a dosing rate of 3.4 kGy/h. The significant enhancement of the EPDM/CIIR blend with 80:20 weight ratio was found suitable for nuclear applications (Ashok et al., 2017). Elshereafy et al. have prepared the HDPE/nitrile rubber (NBR) thermoplastic elastomer blends with different concentrations. The prepared HDPE/NBR blends were then gamma irradiated using a varying dose range from 50 to 250 kGy. The effect of radiation on blend crosslinking was examined. The higher amount of HDPE in the blend at 250 kGy radiation dose resulted in higher gel content and mechanical properties. It was also found that the heat shrinakability of the blends was improved up to 60% when irradiated to a higher gamma dose (Elshereafy et al., 2012). Deepalaxmi et al. have studied the effect of gamma and electron beam radiation on EPDM/silicone rubber (SIR) blend for cable and wire applications. Their study revealed the effect of gamma and electron beam irradiation on the 50–50 composition of SIR-EPDM blend, which irradiated to 25 Mrad dose. The gamma radiation induced crosslinking of the EPDM/SIR blend resulted in improved electrical properties such as surface and volume resistivity and enhanced tensile strength and hardness (Deepalaxmi and Rajini, 2014a). Abdel-Aziz et al. have developed the crosslinked EPDM/LDPE polymer blends and varied the LDPE amount up to 400 phr. This prepared blend combination was subjected to gamma radiation doses. Varying doses of gamma radiation analyzed the effect on mechanical and thermal properties of the EPDM/LDPE blend. The doses were employed from 50 to 500 kGy. They have revealed that increasing the amount of LDPE in the blend system with optimized gammas radiation dose resulted in higher tensile properties and can be useful for automotive applications (Abdel-Aziz et al., 1992).

## 4 Gamma Radiation Resistant Polymeric Systems

Polymer blends are widely used on an industrial scale to add valuable properties of one polymer to another polymer on a compatibility level. Gamma radiation in controlled dose also establishes the interface compatibility in incompatible polymer blends. Gamma crosslinking depends on polymer morphology, where the amorphous phase shows a high formation of the crosslinked network compared to the crystalline phase. Since the amorphous phase has a random, coiled structure and free mobile segment, which enables the matrix for ionization and free radicals among chains that recombine to generate a 3D crosslink network.

Elastomer shows a high degree of crosslinking among chains compared to thermoplastic since elastomer shows high amorphousness compared to crystalline thermoplastics. The crosslinked polymer blends also show high chemical, environmental and thermal stability with superior mechanical strength. The radiation resistance of grafted polymer, polymer blend, and composites systems is generally analyzed by the half-value dose with respect to the mechanical properties. The radiation resistance of polymer is an important parameter. It can be defined as retaining 50% of mechanical properties, especially % elongation, compared to the original one after radiation exposure. The mechanical properties, including tensile, flexibility, and elongation, should not be reduced below 50% at a higher gamma radiation dose, and if not, the polymer can be considered radiation resistance polymeric systems (Wündrich, 1984). In some applications, such as nuclear or space applications, polymeric components are exposed to different radiation environments. In nuclear applications, polymer products face long-term gamma radiation during end-use applications. The gamma exposure for the long term leads to degradation in the polymer chain, making the product brittle and useless with decay in mechanical and thermal properties. People have studied gamma radiation resistance for various polymeric systems. Deepalaxmi et al. studied the irradiation of silicon and EPDM blend up to 250 kGy and analyzed the improvement in the electrical properties. They have found that optimized cross-linking of silicon and EPDM blends at higher irradiation doses occur without any degradation of the molecules. They have also confirmed that the blend showed higher volume and surface resistivity at higher gamma irradiation but reduced elongation. The electron beam irradiated blend system had lower properties as compared to gamma irradiated when applied to the cable insulation applications (Deepalaxmi and Rajini, 2014b). The performance of EVA copolymer depends upon its vinyl acetate (VA) content. The gamma radiation resistance has been studied for EVA/EPDM blends, and the effect of radiation dose and vinyl acetate content on the resistance of EVA/EPDM blends has been reported. As VA content increases, the amorphousness in EVA increases. The coiled and randomness of the EVA chain may enable the EVA to sustain itself in a gamma radiation environment. Ethylene-propylene diene elastomer (EPDM) is widely studied for high energy environments. It has been observed that the coiled and amorphous chain of EPDM can crosslink and sustain gamma radiation exposure for the long term exposure (Ray Chowdhury et al., 2016; Sharma et al., 2018). Moustafa et al. have carried out high gamma dose irradiated polymeric elastomer blends consisting of natural rubber and styrene butadiene rubber. The vulcanization of these various prepared proportions of blends has been carried out at 250 kGy gamma radiation dose. The enhancement in the tensile modulus and tensile strength was observed. They have also confirmed that the thermal stability of the high gamma irradiated vulcanized blend was higher than that of natural rubber and styrene butadiene rubber (Moustafa et al., 2016). The amount of VA controls rheology, crystallinity, mechanical, thermal, and electrical properties, and flame retardancy of the EVA molecules. EVA has a suitable barrier property, low-temperature excellent toughness, stress crack resistance, ultraviolet radiation, weather resistance, and excellent mechanical properties. However, ethylene–propylene diene elastomer (EPDM) has the high crosslinking ability, excellent heat resistance, ozone resistance, impact strength, and flexibility, which is a perfect material in many areas such as wire and cable insulation, automotive, O-ring, gasket, etc. (Wündrich, 1984). In gamma radiation exposure, plastics and elastomers face ionizing gamma radiation for a long time. They should retain valuable properties such as strength, extensibility, degradation stability, dimensional stability, electrical insulation, etc. (Clough, 2001).

## 5 Preparation of Functionalized Polymeric Compatibilizer *via* Gamma Irradiation to Compatibilized Polymer Blends

Immiscible polymer blends can be compatible by using polymer functionalization, and this consideration can be important in increasing polymer properties for high temperature and radiation techniques (Botros, 2002; Tamada and Maekawa, 2010; Cao et al., 2011; Plummer et al., 2019). Compatibility is a specific interaction between two different polymer phases that reduces the free energy of physical or chemical interaction. Polymer compatibility reduces interfacial tension between two different phases and allows for finer dispersion of one phase to another polymeric matrix phase (Sammler et al., 1992; Klinshpont et al., 1994; Souza and Demarquette, 2002; Retsos et al., 2004). Using the gamma radiation technique, Sonnier et al. developed the compatibilization of two types of immiscible blends. They created HDPE/ground tyre rubber and PP/HDPE polymer blends by varying irradiation from 0–100 kGy (Sonnier et al., 2021). Most functionalization techniques have been used to establish polymer compatibilities, such as conventional chemical grafting on polymer backbones, such as maleic acid, maleic anhydride, glycidyl methacrylate (GMA), and acrylamide, which acts as a compatibilizer in the blending process (Dharmarajan et al., 1995; Chowdhury and Sabharwal, 2011; Mira et al., 2017; Mahendra et al., 2019).

In contrast with chemical grafting, radiation-induced grafting is a useful and popular technique to graft the organic functionalities on the polymer chain. Irradiation also helps to reduce the toxic ingredients used for polymer compatibilization. Gamma radiation grafting is most widely used for the grafting process at a low dose (1–3 kGy). This method is better than physical or thermal induced chemical reactions because it is less hazardous, less toxic, more environmentally friendly, and requires less time (Hama et al., 2010; Davenas et al., 2002). Ajit Singh also discussed the effect of the irradiation process for the compatibilization of polymer blends. He has covered the radiation process required for the polyolefin polymeric blends compatibilization. When organic systems are exposed to air, they oxidize, resulting in the formation of peroxy, hydroperoxy, hydroxyl, and carbonyl groups. This functionalization is then used to make miscible polymer blends (Singh, 2001).

## 6 High Performance Applications

### 6.1 Gamma Radiation Processed Hydrogel for Medical and Biomedical Applications

Gamma radiation induced polymer hydrogels have been used in medical devices includes implants, injectable formulations, dressing materials, assays used for diagnostic, etc. The advantage of using gamma radiation-induced polymers in medical and biomedical applications is that no chemical ingredients are used to initiate the reaction. These polymers and medical devices can be used at any temperature range but only restricted to the surface. In Indonesia, the Centre for Application of Isotopes and Radiation Technology BATAN has prepared the PVP/PVA polymeric hydrogel for the dressing application. Hydrogels have complex polymeric networks that can keep the water inside the open spaces between the polymeric chains. The prepared hydrogel has characteristic benefits: not toxic nature, sterile, water intake of about 80%, better adhesion to the wound, and ease to remove from wound place (Darwis et al., 2015). In another example, hydrogels are used in stimuli-responsive materials, hybrid organs, and implants. Polyvinyl alcohol (PVA) or crosslinked collagen have been used to prepare the injectable polymers. The use of polyHEMA for ophthalmic application has gained importance along with radiation-induced hydrogel such as polymethacrylic acid, chitosan, polyvinyl pyrrolidone, etc. (Rosiak, 1995). The use of ionizing radiation in the fabrication of human-friendly commodities appears to become the most beneficial route of broadening the range of commercial applications of radiation technology.

### 6.2 Radiation Processed Polymer Blends and Composites for Industrial Applications

Radiation is generally used for grafting, curing, crosslink network, and degradation of polymeric components. Radiation technology has benefits over chemical induced polymer crosslinked structure. The main application of radiation-induced polymer includes wire and cable insulation, heat shrinkable polymers, plastic foams, gaskets and seals, polyethylene pipes, moulded engineering products, and hip and knee joint components, prevulcanized radial tyres, etc. (Keizo Makuuchi, 2012). Lawton et al. have motivated Japanese scientists, and prepared radiation induced crosslinked polymers for wire and cable applications and heat shrinkable tubing (Lawton et al., 1954). They have prepared the crosslinked polyolefin in the absence of chemical additives. Furthermore, the irradiation crosslinked polymers were used for plastic foams and tyre component applications. In the 20th century, gamma radiation polymers gained importance in automotive sectors to reduce carbon emissions and lightweight products. In Asian countries, radiation crosslinking of Polytetrafluoroethylene (*PTFE*) has been synthesized and used in several industrial applications. The radiation induced PTFE has higher temperature radiation stability as well as mechanical stability and has been used in high performance applications (Sun et al., 1994). Cleland et al. have used radiation cured thermoset based sheet moulding compound (SMC) composites for the automotive industry. They have replaced the metal and steel parts of the vehicles with radiation cured SMC composites. The replacement of the metal, aluminum, and steel parts also result in the vehicle’s weight loss, increasing fuel efficiency (Cleland et al., 2020).

## 7 Electron Beam Versus Gamma Radiation Processed Polymers

Last 2 decades, radiation technology is being used extensively, and it has become a prevalent and valuable technique for radiation crosslinking of polymers. However, recently, the market of electron beams has rapidly grown-up compared to gamma irradiation due to some limitations of gamma irradiation, e.g., difficulties in large-scale irradiation, time consumption, safety towards handling of isotopes, etc. The electron beam has been widely used for wire and cable, surface modification, crosslinking, and sterilizations. The electron beam has been well accepted compared to gamma radiation since it has less irradiation time, rapid, fast, and high space for irradiation compared to gamma irradiation.

Electron beam radiation is generated using a high energy electron machine. In the E-Beam system, the energy penetration is partial, making it useful for low density products. The E-Beam radiation required significantly less time (deliverance time) as compared to gamma and X-ray, which is suitable for sensitive products (Fifield et al., 2021). The E-Beam radiation technique has gained more importance in polymer sterilization than the gamma radiation technique in recent advancements. E-Beam radiation has rapidly developed attention due to its faster dose deliverance time, short processing time, higher output, higher effectiveness, less cost, and availability of high energy machines. Choi et al. have carried out a comparative study of gamma and E-Beam irradiation on the decomposition of carboxymethyl cellulose. They have analyzed the gamma ray from cobalt-60 and E-Beam radiation with 10 MeV energy on the rheological properties of carboxymethyl cellulose. They have confirmed a drastic decrease in viscosity when exposed to gamma radiation (Choi et al., 2009). Chowdhury et al. studied the ethylene vinyl acetate (EVA) and EPDM blends. They have used E-Beam irradiation to crosslink the blends system. They have also studied the effect of different concentrations of EVA and E-Beam radiation on the blend system’s mechanical, flame resistance, and thermal properties. The irradiation dose was varied from 50 kGy to 150 kGy to analyze the effect on blend crosslinking (Ray Chowdhury et al., 2016). Sharma et al. have carried out the effect of E-Beam irradiation onto the HDPE/EPDM blend. They have fabricated the 2 mm thick blend samples and then irradiated them using high electron beam energy with a 2 MeV E-Beam accelerator. The dose rate in the range of 50 kGy–150 kGy was used to crosslink the blend system. The effect of increasing E-Beam irradiation on mechanical, flame resistance, thermal and morphological properties was characterized (Sharma et al., 2017). Chowdhury et al. investigated the electron beam irradiated polyolefins blend system. The crosslinking of the LDPE/EPDM blend has been studied in the absence of a crosslinker. The prepared crosslinked system can be used in o-rings and gaskets applications. They have discovered that the crosslinking increases with increasing EPDM amount and E-Beam doses, which results in the higher mechanical, thermal, and flame resistance properties of the LDPE/EPDM blends system (Chowdhury et al., 2015). It can be seen form comparative study of E-Beam and gamm radiation, the processing time requiredto cure polymer system usinf E-Beam is less which enhancec its output rate and requird less energy as compared to gamma radiation. Currecntly these E-Beam system have been rapidaly used in the medical, autimitove, industrial and engineering applications.

## 8 High Energy Radiation Processed Polymers: Limitations, Challanges and Future Prosspects

The high energy radiation processed polymeric components show high impact for property devlopment for long erm sustainability and durability. The improvement in mechanical, thermal stability, chemical resistance, electric insulation and environmental properties enable the polymeric product to sustain for long term end use applications (Dawes et al., 2022). However, there are some limitations and drawbacks of high energy radiation processing of polymeric materials which challange the reliability of processing techniques, material performance and processing parameters. As gamma ray possesses high energy (1.2 MeV) which generates instant free radical sites at polymer backbone to result into chain crosslinking and bond scission. Gamma radiation has high penetration power so can be used to sterilize the large amounts of materials of high dense packing. The chain crosslinking improves all application based polymer properties while chain scission deteriorates them. The high energy gamma processing of polymers depends upon various factors such as dose level, dose rate and radiation soure and its half life, chemical structure of polymer matrix, physical state, morphology and thickness of polymer product which decide the alternations in polymer properties and performances (Clough L. Roger; Clough, 2001). The radiation processing of polymeric product is also limited to required degree of crosslinking in matrix which specifies the particular end use applications. Also gamma radiation chamber possess limited space and slow processing speed. However on large scale industrial application, irradiation requires higher space and volume with processing in less time.

The gamma sterilization of biomedical based products has been found to generate important issue for end use applications. Polymeric material after sterilization shows radiation-induced degradation which leads to discoloration followed by decay in end use properties. The high energy radiation processing also faces the challenge of type of molecular structure of polymer backbone. Polymers having crystalline structure e.g. low density polyethylene (LDPE), high density polyethylene (HDPE), and Polypropylene (PP) usually are not likely to go beyond certain limit of dose of radiation i.e. 100 kGy. After some irradiation dose polymer structure shows discoloration and deterioration of molecular structure (Dawes et al., 2007; Reichmanis et al., 1993). While amorphous polymers shows high level of crosslinking and sustainability towards high radiation dose and exposure period. Gamma processing also has been reported to face the radioactive decay in radioactive source (Cs-137 or Co-60) which might be issue for human health and environment.

To overcome the above issues of high energy gamma radiation, industry and researchers have been showing interest towards electron beam (EB) processing for safe, high speed, environmental friendly and high volume processing capacity since last decades. The electron beam irradiation of polymeric products has proved itself at reliability level for an industrial scale processing. However atmospheric condition has also impact on the both radiation processing as presence or absence of oxygen influneces the degree of crosslinking and chain scission in polymer matrix. In presence of oxygen, polymer matrix radicals will form the peroxide and hydroperoxide which lead to rate of chain scission in polymer matrix.

The high energy radiation (gamma and electron beam) processing have opened the door for opportunities for wide areas of applications. The reliability and durability of radiation processed polymeric prodcuts depend upon their mechanical strength, thermal stability, electrical insulation, chemical and environmental resistance (Clough, R., 2001). Due to better property development, the future possibilities and scopes for gamma and electron beam irradiation of functionilzed polymer, polymer blends and composites, nanomaterial and polymeric nanocomposite have been risen up for commercialization e.g. electrical insulation, biomedical sector, tissue engineering, implateble surgical devices, hydrogel, high temperature and radiation resistance polymer material, automobile sector, defence sector and high energy space applications since last decade (Clough, 2001). The gamma irradiation at low controlled dose has been known as popular process for monomer grafting on polymer backbone and strerlization of polymer surgical devices. However relationship between the structural composition of polymeric materials and their irradiation ability with optimization is an imporatnt to apply the right materials for radiation sterilization purpose.

The tuning between crosslink ability and chain degradation of polymer matrix should be also an important scope for to select right radiation processing technique for desired applications.

Importantly, the effect of radiation processing on polymer properties has significant impact for the wide areas of applications such as heat-shrinkable tubes, wire and cable insulation, curing of liquid polymer, plastics, radiation lithography, radiation cured polyethylene based elastomer for solar photovoltaic module encapsulant, curable paints and inks, defence, and outer space applications.

## 9 Conclusion

From this review, it can be concluded that gamma irradiation has a significant impact on modification and improvement in properties of different polymeric systems such as individual polymer, polymer blends, composites, and nanocomposites. The gamma radiation processing polymers have benefits over chemical processes as no additives are necessary for the reaction, can be used at any temperature range applications, well controlled grafting and crosslinking. The gamma radiation modified polymers have found the high end applications in automobiles, film packaging, electrical insulation, hydrogel, tissue engineering, sterilization of medical devices, biomedical and nuclear applications, etc. Apart from the crosslinking and grafting, gamma irradiation can be used for functionalization, sterilization, and compatibilization of various polymeric systems. For compatibility in polymer blends, a low controlled dose of gamma radiation has become a widely useful and most applicable technique to synthesize radiation induced grafted compatibilizer. Gamma radiation processed hydrogel has become the most applicable and significant biomaterial for biomedical and healthcare applications. In this review paper, we have also discussed the radiation resistance polymeric blend and composite system. However, in the last decade, the electron beam (EB) processing technique has been widely accepted on an industrial scale for modification of polymeric components at a large scale with high speed for wire and cable insulation, heat shrinkable tube, ink and paint curing, automobile, gasket, seal, o-ring curing, film food packaging and high temperature applications.

## Abbreviations

DSC, Differential scanning calorimetric; EB, Electron Beam; EPDM, Ethylene-Propylene diene elastomer; EVA, Ethylene-Vinyl acetate co-polymer; GR, Gamma radiation; LDPE, Low density polyethylene; MAA, Metha-acrylic acid; PP, Polypropylene; POE, Polyolefin elastomer; SEM, Scanning electron microscope; TEM, Transmission electron microscope; TGA, Thermogravimetric analysis; WAXD, Wide angle X-ray diffraction.
